# Phenomenological approach to eating disorders: a scoping review

**DOI:** 10.3389/fpsyg.2025.1547214

**Published:** 2025-04-30

**Authors:** Eduardo Barreto Fernandes Silva, Monalisa de Fátima Rodrigues Pimenta Teles, Nayana Leão e Silva de Castro, Camila Pereira de Souza, Lucas Guimarães Bloc

**Affiliations:** Postgraduate Program in Psychology, University of Fortaleza (UNIFOR), Fortaleza, Brazil

**Keywords:** eating disorders, eating and feeding disorders, phenomenology, phenomenological psychopathology, scoping review

## Abstract

Eating disorders are defined as persistent eating disturbances that compromise physical health and psychosocial functioning. Many of the patients transition between different diagnostic categories and have high mortality rates, which highlights the complexity of these illnesses. Using scientific literature, this study aimed to identify the contributions of the phenomenological approach related to eating disorders. A total of 30 articles were selected for analysis after adopting eligibility criteria that included: studies with effective publication status; published between 2013 and 2024; and available in Portuguese, French and/or English. The collection revealed a significant predominance of discussions about anorexia nervosa, followed by binge eating and bulimia. Through analysis, the following discursive themes were listed: corporeality; identity and relationship with others; proprioception and eating behavior; and clinical treatments. It is concluded that these findings highlight a need for treatment perspectives to consider the bodily experience in patients of eating disorders and its relations with otherness and oneself. It is considered that the modes of being in the diagnoses of Pica, Rumination and ARFID should be taken account in further research, as such works could elucidate questions on the nature of eating disorders.

## 1 Introduction

Discussions about Eating Disorders (ED) have become more important in recent decades. The prevalence of diagnosed cases has been increasing, and the mortality and suicide rates associated with these clinical conditions are among the highest in the field of mental health (Galmiche et al., 2019; Miskovic-Wheatley et al., 2023). Eating disorders can be defined as persistent disturbances in eating or related behaviors, characterized by altered food consumption that significantly compromises the individual’s physical health or psychosocial functioning (American Psychiatric Association, 2023). They have a multifactorial etiology resulting from the interaction of biological, psychological, genetic, social, cultural, and historical factors. These factors can be classified as predisposing, precipitating, and maintaining eating disorders (Oliveira and Hutz, 2010; de Stefani et al., 2023).

This definition, based on the DSM-5-TR, considers important biological and psychological aspects, but minimizes the importance of the subjective nuances of the relationship between the subject and their illness. In addition, many patients move between diagnostic categories over time, which indicates the need for a better understanding of the possible common axes in the experience with EDs (Fairburn et al., 2003; Mancini and Esposito, 2021).

As an alternative to this admittedly nosographic perspective, the phenomenological approach is suggested as a possible way of understanding those who suffer from EDs. In addition to a philosophical perspective, phenomenology has been used as a way of articulating and understanding the experiences of illness, whether in the field of phenomenological psychopathology (Mancini and Esposito, 2021; Spencer et al., 2024), by presenting a descriptive and comprehensive approach to the different ways of existing, or in the construction of research models that use the empirical phenomenological method. In this article, the use of the term ‘phenomenological approach’ indicates a scope that includes the recognition of the structures of subjective experience in eating disorders from a theoretical point of view, through articulations with elements of phenomenology, and also by way of demarcating the field of empirical research that has as its center the use of the phenomenological method as a way of accessing the lived experience.

The main axis of the contemporary phenomenological approach to EDs is phenomenological psychopathology. This is a field of knowledge that originated as an offshoot of 20th-century psychiatry and was initially established as a means of dialogue between psychiatry, psychopathology, and philosophical phenomenology. From this perspective, contemporary authors have sought to understand the common axes of the experience of EDs based on phenomenological themes that involve the relationship with the body itself, intersubjectivity, identity, and the space and time lived by these patients (Castellini et al., 2014; Mancini and Esposito, 2021; Stanghellini et al., 2019; Stanghellini et al., 2021; Tarchi et al., 2024).

Therefore, the need to conduct this scoping review of the scientific literature in this area is justified due to its depth and particular contribution to the theme of EDs. It is understood that gathering data on the contribution of the phenomenological approach to EDs can contribute to the development of clinical interventions that take into account the experience, beyond the symptoms, of those who suffer from them (Stanghellini et al., 2021). This article aims to identify, in the scientific literature, the contributions of the phenomenological approach to EDs in scientific productions published until the year 2024.

## 2 Method

The scoping review that underpins this study was carried out based on an original research protocol, using the guidelines proposed by the Manual of Evidence Synthesis, published by the Joanna Briggs Institute. The facilitating model used to structure this research comes from the Preferred Reporting Items for Systematic reviews and Meta-Analyses (PRISMA) guide, specifically its extension for scoping reviews (PRISMA-ScR) (Tricco et al., 2018).

Adapting from these sources, the elaboration of the scoping review is carried out in seven main consecutive steps: (1) definition of the guiding question and objective of the research; (2) search for relevant sources and databases that enable the purpose of the review; (3) selection of studies according to the criteria defined by the research protocol; (4) data mapping, based on reading the studies and identifying relevant information; (5) synthesis of information and summarization of results; (6) analysis of the synthesized information in order to identify patterns in the literature; and (7) presentation of the results, providing an overview of the literature and identifying possible gaps in the research (Arksey and O’Malley, 2005; Peters et al., 2020). Thus, as a triggering question for this collection, the following question is asked: How does the phenomenological approach contribute to the understanding of eating disorders nowadays?

### 2.1 Research strategy

The following scientific portals were used to collect data: CAPES, PUBMED and EBSCOhost. These domains were chosen due to their nature as portals with different databases inserted. During preliminary searches, they provided a wide range of results, which made it easy to eliminate duplicate results in the databases. In the research carried out, the following inclusion criteria were considered eligible: only those with effective publication status; published from 2013, the year of publication of the DSM-5, until November 2024; published in Portuguese, French and/or English; and responding to the keywords.

Given the broad scope of this review, the first phase of the research used each respective ED described in the DSM-5 as a keyword, in association with the Boolean operator “AND” and the keyword “Phenomenology.” Thus, the structure of each research descriptor was as follows: “Phenomenology” AND (“Eating Disorder” OR “Eating Disorders” OR “Bulimia” OR “Anorexia Nervosa” OR “Binge Eating” OR “Avoidant/Restrictive Food Intake Disorder” OR “ARFID” “Pica Disorder” OR “Rumination Disorder”). This research was conducted in two instances for each database, with descriptors in Portuguese, French and English. This stage took place between August and November 2024.

Works related to diagnoses that address ED from a medical or purely nutritional perspective and ED in children were excluded from the review. These were not incorporated due to the degree of complexity regarding the elaboration of the diagnosis and treatment. Similarly, works that do not discuss ED through a phenomenological lens, whether philosophical and/or methodological, are outside the scope of the review. Temporal filters were used to include only articles published from 2013 to 2024.

### 2.2 Evidence selection

The articles collected in the first stage of the research were entered into the EndNote Online Classic citation management platform (Clarivate, 2024), where all detected duplicates were excluded. Thus, 4,063 articles were initially identified (EBSCOhost: 2,643; PubMed: 360; CAPES Portal: 1,060) based on the descriptors used in all languages. After applying temporal and publication status filters and eliminating duplicate articles, 445 articles were kept (EBSCOhost: 246; PubMed: 86; CAPES Portal: 131) for later analysis.

**Figure 1:**
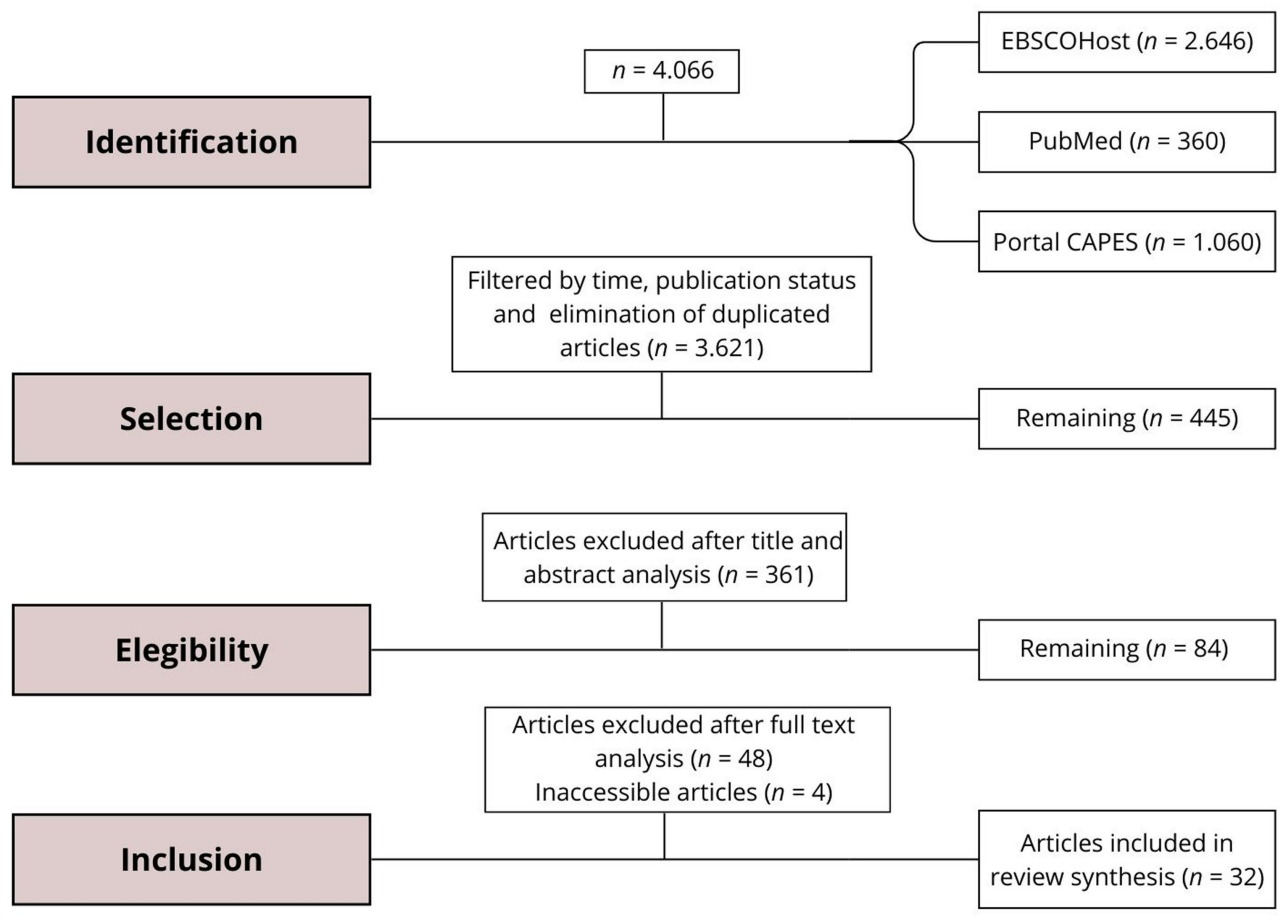
Flowchart of study identification and selection.

For the second stage of the research, filtering was performed based on the titles and abstracts of the remaining articles. This reading was carried out by three independent evaluators and, taking into account the criteria and scope of the review, those considered suitable were attached to a separate folder on the EndNote Web platform. After this screening, 84 articles were identified as suitable for a complete analysis. The selected articles were subsequently read in full, with the exception of 4 which were inaccessible to the authors. After reading, 32 articles were accepted for inclusion, and their metadata and contents were synthesized for use in this review. Of these data, the following were highlighted: the diagnosis evidenced; the philosophical basis; research method; summary of the theoretical discussion; psychological perspective; publication period; and language and authors. The remaining 48 rejected articles were not considered suitable because they did not consider a phenomenological perspective in the etiology of ED, despite many using phenomenological research methods in their preparation (see Figure 1).

## 3 Results

The prevalence of studies that use the diagnosis of anorexia nervosa as part of their main discussion is evident, encompassing 17 of the 32 studies selected. In addition, we found 1 study that presents the diagnosis of bulimia nervosa and 4 studies that discuss binge eating, sometimes under the name of “hyperphagia.” Finally, 10 publications discuss the diagnostic category of eating disorders as a whole. As expected, the vast majority of studies were presented in English, totaling 24 articles, while French and Portuguese were represented by 6 and 2 studies, respectively. No study used the diagnoses of Avoidant/Restrictive Food Intake Disorder (ARFID), Pica Disorder or Rumination Disorder as the core of their discussion in any of the languages listed.

As illustrated in Table 1, the studies collected will be referred to hereinafter by their numbering. The works identified were mostly discussions on ED from the field of Phenomenological Psychopathology, comprising 17 articles (2, 8, 9; 11 to 13; 17 to 19; 21 to 25; 27, 28, and 30). They were written by professionals in the field of psychiatry and/or psychology, with a greater focus on clinical case studies and theoretical discussions. The others presented discussions at the core of Existential Phenomenology, collaborating with exploratory productions from various fields of knowledge; articles 16 and 29 are works by professionals in Physiotherapy, 14 is from the field of Anthropology with theoretical frameworks in linguistics, while 3 and 20 are publications by public health agencies, with no connection to psychological practice beyond the study of diagnostic categories. The other studies (1; 4 to 7; 10, 15 and 26), although they involve clinical treatment, present more comprehensive philosophical discussions, finding foundations in classical texts and other articles of a similar nature. The relationship between articles 4, 5 and 7 is notable, as they are theoretical essays that respond to study 6: “Anorexia Nervosa and the Body Uncanny: A Phenomenological Approach” (Svenaeus, 2013a,b).

**Table 1:** Main characteristics of the selected articles.

N°	Title	Country (Language)	Reference (Authors, Year)
1	Is Anorexia Nervosa a Passion?	English	Bowden (2013)
2	Fenomenologia do transtorno do comportamento alimentar hiperfágico e adicções	Portuguese	Charbonneau and Moreira (2013)
3	No rastro do que transtorna o corpo e desregra o comer: os sentidos do descontrole de si e das “compulsões alimentares”	Portuguese	Nunes and Bittencourt (2013)
4	Anorexia: A Disease of Doubling.	English	Leder (2013)
5	Anorexia: Beyond the Body Uncanny.	English	Morris (2013)
6	Anorexia Nervosa and the Body Uncanny: A Phenomenological Approach	English	Svenaeus (2013a, 2013b)
7	The Body As Alien, Unhomelike And Uncanny: Some Further Clarifications	English	Svenaeus (2013a, 2013b)
8	Anorexie et intersubjectivité: étude phénoménologique et éthologique	French	Englebert (2015)
9	From Body Image to Emotional Bodily Experience in Eating Disorders	English	Gaete and Fuchs (2016)
10	On judgement day: Anorexic and obese women’s phenomenological experience of the body, food and eating	English	Moola and Norman (2017)
11	Phénoménologie du vécu hyperphagique dans l’obésité: le corps, le monde et l’autre.	French	Bloc et al. (2018)
12	The body and the other: a crisis of the self-representation in the disorders of post-modernity	English	Castellini (2017)
13	Anorexia Nervosa and First-Person Perspective: Altruism, Family System, and Body Experience	English	Englebert et al. (2018)
14	Beyond the clinic? Eluding a medical diagnosis of anorexia through narrative	English	Shohet (2018)
15	À propos d’un trouble de l’évidence corporelle dans l’anorexie	French	Wykretowicz et al. (2019)
16	Patients’ experiences from basic body awareness therapy in the treatment of binge eating disorder-movement toward health: a phenomenological study	English	Albertsen et al. (2019)
17	Anorexie mentale et trouble du comportement alimentaire selon une perspective phénoménologique: version francophone du questionnaire IDentity and EAting disorders (IDEA)	French	Englebert et al. (2019)
18	Abnormal time experiences in persons with feeding and eating disorder: a naturalistic explorative study	English	Stanghellini and Mancini (2019)
19	The Pathogenic and Therapeutic Potential of the Gaze of the Other in the Clinic of “Eating Disorders”	English	Esposito and Stanghellini (2020)
20	Embodying Experiences with Nature in Everyday Life Recovery for Persons with Eating Disorders	English	Jepsen Trangsrud et al. (2020)
21	Emotional depersonalization in persons with feeding and eating disorders	English	Mancini and Stanghellini (2020)
22	Body experience, identity and the other’s gaze in persons with feeding and eating disorders	English	Stanghellini and Mancini (2020)
23	Bridging cognitive, phenomenological and psychodynamic approaches to eating disorders	English	Castellini et al. (2022)
24	The disappearing body: anorexia as a conflict of embodiment	English	Fuchs (2022)
25	Lived body and the Other’s gaze: a phenomenological perspective on feeding and eating disorders	English	Mancini and Esposito (2021)
26	Controlling the Noise: A Phenomenological Account of Anorexia Nervosa and the Threatening Body	English	Osler (2021)
27	The role of embodiment in the treatment of patients with anorexia and bulimia nervosa: a 2-year follow-up study proposing an integration between enhanced cognitive behavioural therapy and a phenomenological model of eating disorders	English	Rossi et al. (2021)
28	L’anorexie mentale: une fatigue de ne pas être soi?	French	Merand and Lemoine (2022)
29	A preliminary exploration of experiences of integrating the body in the self in two women with anorexia nervosa in view of phenomenological conceptualisations	English	Naess and Kolnes (2022)
30	La tanière et le territoire: esquisse d’une topo-analyse des troubles du comportement alimentaire	French	Saraga and Wykretowicz (2022)
31	Anorexia nervosa through the lens of a severe and enduring experience: ‘lost in a big world’	English	Kiely et al. (2024)
32	Reframing Anorexia Nervosa: A Phenomenological Exploration of the Self-Other Relationship with Husserl’s Intersubjective Theory.	English	Zhang (2024)

Repetition of authorship in the productions is expected given the scope of this review. The authors Stanghellini (articles 17, 18, 19, 21, 22, 23, 27) and Mancini (17, 18, 21, 22, 23, 25, 27) are credited in 7 productions (21.8%), while Englebert (8, 13, 17) and Castellini (12, 17, 23) appear in 3 works (9.3%), Svenaus (6, 7), Fuchs (9, 24) and Wykretowicz & Saraga (15, 30) have 2 publications (6.2%), and the other authors have 1 publication (3.1%) incorporated in this review.

The most recurrent themes among the studies were divided into the following axes, namely: (1) Loss of Bodily Evidence; (2) Identity in the intersubjective relationship with the other; and (3) Proprioception and Eating Behavior. Also noteworthy are the ideas related to (4) Clinical Treatment, focused on the resolutions reached on the therapeutic stance through the phenomenological lens.

### 3.1 Loss of bodily evidence

Although all productions address corporeality, some works (11, 15, 17, 24, 25 and 29) highlight bodily perception as a key criterion for theoretical discussions and etiological studies. They indicate a change in bodily experience as a fundamental axis in ED.

Some cases portray a reality in which the demands of the biological body exceed the individual’s capacities for meaning, resulting in pathological experiences as evidenced in study 11 by the use of the expression “short-circuit” of the bodily experience. This illustrates a lack of stable connection between experiential reality and that of the physical body, which results in binge eating.

The results of study 25, which corroborate the discussion above, elaborate on the hypothesis of an optical-kinesthetic disproportion, proposing that this alteration originates from a field of signification, in which the gaze on the body appears hypertrophied as a response to the decrease in significant kinesthetic experience. The authors use results based on neuroanatomical studies with anorexic patients, but their rationale warns about the common axis of corporeality in eating disorders. Similarly, study 29 points to a loss in image and kinesthetic intelligence in patients with anorexia, corroborating the use of this hypothesis in treatment.

The sensation of absence or total disappearance of the body is found as a discursive link in findings with patients with anorexia, mainly in analyses of self-reports and case studies. Studies 15 and 24 use, respectively, these methodologies and present the idea of a loss of bodily evidence at the core of their discussions; that is, a loss of intersubjective contact with the body, whether perceptive or emotional, which has repercussions on communication with others and with the world, since the body is the mediator of our lived experiences.

In addition, there are lines of discussion that point to other possible experiential vulnerabilities resulting from ED. In a case study, article 8 points to a possible weakened axis in intersubjectivity, through which the relationship with food is revealed. The author seeks to point out the foundations of the anorexic being-in-the-world, and identifies them in patterns of functioning that they refer to as altruistic.

### 3.2 Identity in the intersubjective relationship with the other

In attempting to understand the axis of intersubjectivity as a foundation for the discussion of the phenomenological approach to ED, studies 1, 3, 4, 12, 13, 19, 22, 25, 28, and 32 discuss how body image and weight appear as a possibility of existence in which the individual seeks to get in touch with their personal identity through an attempt to control their emotions, their individuality and their relationship with the world and with others. For a global understanding, it is imperative to consider issues such as the individual’s perception of their own body, the connection with the perception of others and how they experience their corporeality.

Study 1 addresses identity in the context of anorexia nervosa by theorizing, based on references in the literature, about the identification made between anorexic people and their disorder, making it central to their understanding of themselves. Different authors and their approaches to conceptualizing anorexia nervosa as an external entity are mentioned, indicating the complex relationship between the individual’s sense of identity and the eating disorder.

This line of theorizing appears to be corroborated by study 25. As previously mentioned, this study discusses a possible weakened axis in the bodily experience and how becoming ill involves deeper changes in experience. Thus, the authors suggest that people with eating disorders perceive their own bodies primarily as objects observed by others due to a disturbed kinesthetic apprehension.

The identity-body relationship is similarly elaborated in study 12, as well as a greater focus on its social implications. For the authors, the disturbed experience of an individual with ED is associated with the process of identity restructuring, in which they encounter difficulties in appropriating their own existence. This study examines the centrality of the body and its relationship with the process of identity construction, especially in the context of eating disorders and gender dysphoria. In this regard, a possible line of dialogue is presented with study 3, which elaborates on feelings of alienation and identity confusion in an individual who experiences a mismatch between the external reality of the body and the subjective internal perception. They experience a daily struggle to establish a relationship between the representation of their body and their body as a real object.

Delving deeper into the relationship of the individual immersed in society, study 13 contributes to the psychopathological understanding of anorexia nervosa by considering the emotional and corporal investment in relationships, as well as the social and cultural dimensions of the act of eating. Moreover, the implications of relationships in the structure of being are addressed by study 32, whose author appropriates Husserl’s transcendental phenomenology. This discusses the pathologized experience based on an experiential asymmetry in regard to the relationship of the corporal and intersubjective axes, appropriating concepts from the phenomenological tradition to explore anorexia.

Narratives about an uncertainty inherent to the bodily experience, as well as the appeal of ED patients for certainty in the subjective axis, are repeated in the studies on this topic. Vulnerability towards the body, however, is discussed in studies 19 and 28 from a functional perspective, creating a narrative about the body as an instrument for creating identity. The authors of article 22 value this relationship in their theoretical discussion, exploring how individuals with ED experience their identity as a mirroring of the vision of others, supported by the Sartrean concept of a “body-for-the-other.” This external gaze becomes the main means by which they understand and define themselves, reflecting a sense of self that depends on the approval or disapproval of others. There is a significant alienation from one’s own body and emotions, and a scenario of strong dependence on external recognition is established.

### 3.3 Proprioception and eating behavior

In studies 1, 2, 9, 11, 21, 26, 31, the emotional and perceptive experience of oneself is discussed in its direct correlation with the eating behavior displayed in the symptomatology of EDs. While the concept of proprioception comes from the areas of neuroscience and developmental psychology, understood as “the unconscious knowledge of where the different parts of the body are at a given moment” (Liutsko, p. 107, 2013), here it is extended to also mean the feelings that are found in the depths of the eating experience and the way they are confused with physiological signals.

The body’s signals and the ways in which they are shown in the experiential field are the basis of the discussion in article 11, already mentioned previously for its dialogue between the existential and perceptual axes of the body. From another perspective, article 26 explains how one of the axes of proprioception for anorexic patients surrounds a feeling of being constantly demanded by the physiological body, here described as “visceral.” This position reveals a radical objectification of the bodily experience, in which the body presents itself as a limitation and restriction of subjectivity due to basic physiological needs, thus creating a narrative of a fundamental lack of control over one’s own actions.

The idea that eating in hyperphagia is permeated by feelings of lack of control is problematized in article 2, which analyzes to what extent it can be considered an addiction. In what approaches this category, the authors argue that compulsive eating has an axis in relieving tension and lack of stimuli, whether due to a nervous or depressive state, to the detriment of a significant engagement of the proprioceptive paradigm that causes it. However, for the authors it would be affectively poor to consider eating behavior only as an addiction, considering the cultural significance given to eating as a platform for social and emotional contact.

When conceptualizing the idea of basal states that allow for an unhealthy experience of eating, article 9 elaborates on body image as a construction of emotional experience. For the authors, the emotional axis is understood as that which places the body beyond the field of consciousness; emotions, which are invariably also physical sensations, are relegated to the “background” of perception on a daily basis and do not dialogue with bodily (dis)satisfaction.

The understanding of the affective experience intertwined with corporeality is corroborated by the authors of article 21, who portray the emotional axis as that which governs the values that move the individual. For them, the different psychopathological conditions, whether EDs or comorbidities with eating disorders, exhibit distinct emotional characteristics, but are internally consistent within each diagnostic category.

In contrast, the perception of eating disorders as the result of a form of misdirected affective investment is contraindicated by article 1. In response to an article entitled “Anorexia Nervosa as A Passion” (Charland et al., 2013), the authors discuss “passions,” that is, the implicit emotions that motivate and maintain behaviors, based on the Heideggerian concept of mood as a manifestation and disposition of being (Heiddeger, 2015, p. 134). This would be the subjective means by which an individual attributes value and existential meanings to the facts of everyday life.

In addition to the emotional experience, article 31 analyzes the production of meanings by a patient with anorexia nervosa in a severe and persistent condition. Thus, the authors perceived recurring themes regarding the weakening of the self dimension, as well as a struggle to establish experiences that go beyond anorexia. In a cyclical movement, the experience of anorexia would trigger and be triggered by processes of dissociation between the sensitive experience and the perception of oneself, so that perceptible damages are established in various aspects of mundane life. Thus, the authors highlight the possibility of approaching the sick dimension based on the concept of a “global impoverishment” of the self and its implications in cases where anorexia is chronic.

### 3.4 Clinical treatment

Although the discussions in each article can be extrapolated to a pragmatic application, it is understood that there is an axis of production whose research methodology seeks to answer questions about the development of an appropriate clinical intervention based on the theoretical contribution of phenomenology (16, 17, 20, 23, 27, and 29).

Elaborating on the discussions about corporeality, articles 16 and 29 use physical movement in empirical studies as a way of exploring the bodily experience of ED patients. In the first article, these patients recovering from binge eating were able to get in touch with physical sensations through Body Awareness Therapy in conjunction with therapeutic monitoring, and their reports at the beginning and end of the process were compared and analyzed phenomenologically. For the authors, a discursive meaning emerges that replaces the idea of a “problem” body, at first, with that of a body as a path of possibilities. The themes that emerge, for the authors, recount a narrative of a body fragmented in its subjective experience and that (re)finds unity based on the experience of the concrete body. Similar narratives emerge in an exploration with anorexic patients in article 29. In this article, the patients underwent a long process of psychomotor physiotherapy treatment, about which they were interviewed. Through analysis, emerging themes were perceived regarding the therapeutic relationship, changes in the way of connecting with the body and improvements in the ways of articulating emotions. The interviewees reported significant perceptions about the way in which the sensory axis of their body is affected by so-called aversive feelings, so that the body becomes a foundation for avoiding contact with these feelings. Moreover, the results obtained in study 20 point to a similar resolution, despite the use of a different methodology. In this study, the patients, people in the process of recovering from EDs, were interviewed in two moments, as in the aforementioned article, but in which the second moment was carried out in direct contact with nature. The narratives emerging from this contact report a relief from psychological stressors, as well as the construction of an awareness of the physiological body through material contact with the environment.

In the field of psychotherapy, the authors of article 27 sought to investigate a possible intervention based on a longitudinal investigation with anorexic and bulimic patients. The study proposes a mediation between a phenomenological perspective on the psychopathology of EDs in conjunction with an interdisciplinary treatment model based on a behavioral intervention, collecting quantitative data over a 2-year period, in order to understand the impacts of therapeutic action on what the authors call a disorder in embodiment. The questionnaires used showed a noticeable decrease in the report of dissatisfaction with the patients’ own images, as well as in the symptomatology under treatment, suggesting the effectiveness of the treatment. However, the authors perceive a remission rate understood as unsatisfactory, as well as a transience between diagnostic categories in a portion of the patients.

Among the instruments used in the aforementioned research is the Identity and Eating Disorders questionnaire (IDEA) (Stanghellini et al., 2012). When applied, the questionnaire seeks to identify psychopathological phenomena related to ED based on responses about concepts commonly experienced by its carriers, such as identity experienced through the body and the perception of the gaze of others. Its validation was carried out and published in article 23, as well as its French-language version in article 17.

## 4 Discussion

Among the themes addressed by the articles, it is clear that the body and the different ways of exploring corporeality appear as a significant and recurring axis. The bodily experience is positioned, within the scope of phenomenological psychopathology, as one of the bases that constitute the intersubjective experience (Messas and Fukuda, 2018; Souza et al., 2020). Thus, the eventual imbalance between the experiences of the body-subject (the body that one is) and the body-object (the body that one has) is the basis for a series of psychopathological experiences, such as those found in EDs (Castellini et al., 2014; Mancini and Esposito, 2021; Stanghellini et al., 2021).

Disturbances in corporeality have been presented as one of the main maintainers of EDs in the literature (Musolino et al., 2020; Rossi et al., 2021; Portingale et al., 2024). For phenomenology, the body is one of the basic dimensions that structure the foundation of human existence and a constitutive element of intentional consciousness (Messas et al., 2018). This perspective helps us discuss the different ways of experiencing corporeality in the different diagnoses of EDs and, thus, understand the profound changes in the foundation that supports them (Bloc et al., 2018).

It is understood that most of the studies that discuss the phenomenology of corporeality do so through the anorexic experience (Fuchs, 2022; Merand and Lemoine, 2022; Moola and Norman, 2017; Morris, 2013; Naess and Kolnes, 2022; Osler, 2021; Rossi et al., 2021; Svenaeus, 2013a, 2013b; Wykretowicz et al., 2019). In fact, despite the discussion of common axes in the body experienced by patients with EDs in general, none of the collected studies show the sensoriality of the body in the experiences of compulsion and bulimia, which would allow for narratives about identity and body image in greater quantity. Phenomenologically, it can be understood that these are the themes that appear in the experiential field of these patients; however, for the authors of this article, this gap is notable. These results seem to heavily suggest that phenomenological research could better contribute with investigations of the bodily experience in less explored EDs, such as Pica, ARFID and Rumination disorders; in turn, lack of meaningful results of this nature could also mean that, while a notable phenomenon, corporeality disturbances may not be the core axis of all ED psychopathology.

In anorexia nervosa, a common axis is found in the incessant control over the body, sometimes described as a result of the loss of bodily evidence (Wykretowicz et al., 2019; Fuchs, 2022). To the extent that the body is the state at which consciousness presents itself in the world, its absence removes the intentionality of contact with other means of signification besides those desired, so that the way in which the body presents itself becomes (almost) solely a choice of the individual.

The intersubjective constitution of the anorexic being-in-the-world reverberates in restrictions on the division of food and appetite with regard to their altruistic relationship with others. Characteristics considered secondary to eating behavior can be viewed from an evolutionary and etiological perspective as an aggrandizement of the social-food needs of others to the detriment of one’s own (Englebert et al., 2018). Thus, one is faced with a paradoxical experience, where the individual is reflexively aware of their own nutritional needs and chooses to relegate them, according to the authors, to “other bodies.”

Discussions about the body also point to it as a constitutive element in the identity of individuals with ED, since their (un)acceptable expressions are traversed through intersubjective contact with others and with the social world. Regarding intersubjectivity in eating disorders, the solidification of existence is understood from the perspective of the other. In addition to social-affective contact, philosophers of the phenomenological tradition, such as Merleau-Ponty (2018) and Sartre (2015), discuss the relationship between Being and an Other, a blurred boundary that organizes existence in the world (Souza et al., 2020; Jacoby and Carlos, 2005). In this way, the perception that the Other perceives one and one’s own perception of oneself are shown to be radically rooted in the world. Thus, as others assign meaning to one’s existence, an individual’s experience is cemented in the world. In a collinear way, it can be said that when assigning an image to a physical and/or bodily appearance, the gaze of the other is fundamental to the social identity of the person being seen. In fact, the absence of a flexible axis of identity incorporation can be a sign of a pathological experience (Bloc et al., 2018). The identity-body-other relationship appears to indicate a path of bodily experience that is strongly dependent on the identity axis, offering an understanding that relates to different aspects of the experience of EDs.

The complex intersubjective dimension of anorexia nervosa stands out, discussed in some findings as excessive care for others or a hyperawareness of oneself. This can be elaborated as an altruistic inclination related to an excessive emotional investment in relationships, so that the Other dominates the field of subjective belonging; the individual experiencing ED would be the “weak link” in relationships, and would depend on their peers to cement themselves intersubjectively (Englebert, 2015; Englebert et al., 2018).

In general, we identified that the authors discuss, in their respective publications, a difficulty of ED patients in tolerating the natural oscillation between experiences of their own body-object and body-subject. Seen primarily as an unreliable, changeable and incomprehensible entity, the body is sometimes seen as an instrument for the constitution of identity in ED due to its capacity for drastic change (Stanghellini and Mancini, 2019; Merand and Lemoine, 2022) or, even, due to its incompleteness (Saint Aubert, 2013). This attribute would be fundamental for the establishment of a “true subjectivity” or the “real” representation of the subject in a physical environment. Thus, there would be anguish in the conception that the other observes him/her, and decides how to value his/her image, regardless of his/her will; controlling this variable would trigger total control over the body. This excessive self-objectification is at the core of the psychopathological experience of ED. Ambiguous narratives are perceived about the embodied identity, which is “captured” by the Other and alienated from the being and, at times, purposefully modified to represent an interiority through an optical path (Esposito and Stanghellini, 2020; Stanghellini and Mancini, 2020; Merand and Lemoine, 2022).

The identity of individuals with eating disorders appears to be closely linked to appearance and social standards, often resulting in a distorted self-image based on the perceived judgments of others. Thus, approval or disapproval expressed through the gaze of others can evoke feelings of pride or shame, reinforcing the individual’s sense of identity based exclusively on external appearance. In short, the interaction between identity and the gaze of others would be a crucial factor in understanding the psychopathological experience of EDs, in which identity is not only constructed, but often distorted by external perceptions. The results on eating behavior in EDs highlight the experience of dysregulated eating as a bodily process, in its physiological and meaningful processes. When the body shows itself in the experiential field through its vital needs, the individual must go through a decision on how to treat it: if they are hungry, they may choose to eat; if they feel uncomfortable with the space they occupy or the shape of their body, they choose not to eat. The extent of this proprioception goes beyond sensory functions, being also a product of personal and social learning of emotions (Gaete and Fuchs, 2016).

Likewise, we start from the perspective of a constant balance between the meaning of “having” or “being” a body, so that eating behavior, whether healthy or sick, is always based on the (mis)appropriation of ways of existing in the world. The same can be said about the value given to experience, namely: in the findings of study 11, the hyperphagic patients interviewed explain the experience of lack of control over emotions that, associated with the dissociation between themselves and their bodies, appear to be translated into an active and bodily eating behavior. In anorexia, an ideological conflict arises through the restriction of food as a way of controlling one’s own subjectivity (Legrand and Taramasco, 2016). The person experiencing compulsion would be someone who experiences a constant weakening of that experience, and the expectation that this state will soon pass is what fosters compulsive action, despite the suffering involved (Nunes and Bittencourt, 2013). Thus, the plurality of affective experience and the ways of exploring it can be gateways to working on the dimensions of the body, as they reference each other. In a therapeutic context, this understanding brings the possibility of a radical deepening of the experience of compulsion. However, the concept of emotions that maintain a symptomatology, although central to the phenomenological discussion of anorexia, does not encompass the explicit narratives that many anorexic patients establish between disordered eating and concepts such as identity (social and personal) and the body (experienced or physiological). The authors of this article suggest that future scientific productions should cover affective issues in addition to those related to the corporeality of EDs.

We highlight the importance of studies on the clinical treatment of EDs, which demonstrate the importance of interventions focused on the dimension of the body intertwined with perception. These can be beneficial beyond a simple change in eating behavior by enabling the resignification of the bodily experience. The data from the articles collected point to the need for a transdiagnostic model for interventions with EDs. Considering the correlation between the intervention for the disorder in the embodiment of these patients, their symptoms and the subjective discomfort reported, it is also understood that there is a relationship that maintains the psychopathology of EDs based on disturbances in the bodily experience, not limited to the category of symptoms alone. The authors suggest that an integration of phenomenological concepts into this treatment model, particularly those related to first-person experience, can contribute to overcoming some of the perceived limitations. Understanding how people with EDs live their experiences (Stanghellini et al., 2021) can contribute to the construction of other, possibly healthier, ways of living their own bodily, relational and identity experiences.

In the case of the IDEA questionnaire (Stanghellini et al., 2012), its theoretical precepts are used in research and validation that reinforce the need for a transdiagnostic and qualified investigation into people with ED in order to understand and intervene in axes that maintain the disordered relationship with eating.

## 5 Limitations of the study

This study is limited in its ability to engage with studies related to the psychopathology of childhood-related eating disorders and their incidence factors. Furthermore, any mention of Pica, Avoidant/Restrictive and Rumination disorders is absent in the time period of the studies’ collection, indicating the possible need to expand the search terms, temporality or databases in future research.

## 6 Final considerations

In light of this study, the need to understand eating disorders through an investigation focused on their pathological experience is reinforced. The results obtained bring to light important phenomena regarding the bodily experience and the social environment that appear to resonate near universally, even though the particularities may differ between each case.

The data collected by this research highlights contemporary advances in the investigation and treatment of certain eating disorders from a phenomenological perspective, whether associated with the field of Psychology or other areas of intervention. The multiplicity of areas of production, associated with the findings that dialogue with each other, seem to point to a common development in the perspective of approach to the treatment of EDs, focused on interventions in regard to the image and understanding of patients’ bodies. Interdisciplinary care for EDs, for example, has been further researched in recent years, and the papers here collected may serve as theoretical common ground between fields working towards the same goal of enabling recovery in ED patients.

While this paper has attempted to provide a comprehensive scope of the current and ongoing phenomenological research, it is an ever growing discipline, especially in the field of Psychopathology. The emphasis on the lived experience of patients, while considered by the authors as a strength, must also be recognized as a part of its limit, in such a way that investigations of pathological experiences may not precisely align with the one described by diagnostics manuals, who mainly focus on dysfunctional behaviors. Thusly, a lack of works which tackle Pica, ARFID and Rumination disorders in the current review may speak of this mismatch; rather than a (sole) underrepresentation of these diagnoses, empirical and theoretical studies may not have yet grasped pathological lived experiences that relate to the symptoms described.

Although the lack of discussions about Pica, Rumination and ARFID disorders can be seen as a weakness of the area, the discussions established throughout this work present a strong theoretical framework regarding the diagnostic category of eating disorders as a whole. Furthermore, patient interviews analyzed by a phenomenological lens, such as through Interpretative Phenomenological Analysis (Eatough and Smith, 2017), alongside the application of questionnaires targeting body image and the lived body experience may shed light into the modes of being in these diagnoses that are still little explored.
